# ACTH Prevents Deficits in Fear Extinction Associated with Early Life Seizures

**DOI:** 10.3389/fneur.2016.00065

**Published:** 2016-05-02

**Authors:** Andrew T. Massey, David K. Lerner, Gregory L. Holmes, Rod C. Scott, Amanda E. Hernan

**Affiliations:** ^1^Department of Neurological Sciences, University of Vermont College of Medicine, Burlington, VT, USA; ^2^Department of Biological Sciences, University of Bath, Bath, UK; ^3^College of Arts and Sciences, Dartmouth College, Hanover, NH, USA; ^4^Institute of Child Health, University College London, London, UK

**Keywords:** epilepsy, cognition, development, prefrontal cortex, melanocortins, ACTH

## Abstract

**Objective:**

Early life seizures (ELS) are often associated with cognitive and psychiatric comorbidities that are detrimental to quality of life. In a rat model of ELS, we explored long-term cognitive outcomes in adult rats. Using ACTH, an endogeneous HPA-axis hormone given to children with severe epilepsy, we sought to prevent cognitive deficits. Through comparisons with dexamethasone, we sought to dissociate the corticosteroid effects of ACTH from other potential mechanisms of action.

**Results:**

Although rats with a history of ELS were able to acquire a conditioned fear learning paradigm and controls, these rats had significant deficits in their ability to extinguish fearful memories. ACTH treatment did not alter any seizure parameters but nevertheless was able to significantly improve this fear extinction, while dexamethasone treatment during the same period did not. This ACTH effect was specific for fear extinction deficits and not for spatial learning deficits in a water maze. Additionally, ACTH did not alter seizure latency or duration suggesting that cognitive and seizure outcomes may be dissociable. Expression levels of melanocortin receptors, which bind ACTH, were found to be significantly lower in animals that had experienced ELS than in control animals, potentially implicating central melanocortin receptor dysregulation in the effects of ELS, and suggesting a mechanism of action for ACTH.

**Interpretation:**

Taken together, these data suggest that early treatment with ACTH can have significant long-term consequences for cognition in animals with a history of ELS independently of seizure cessation and may act in part through a CNS melanocortin receptor pathway.

## Introduction

Psychiatric comorbidities and cognitive deficits are common in pediatric epilepsy. Children with epilepsy are at increased risk for depression, obsessive–compulsive disorder, anxiety, ADHD, autism, significant difficulty with learning and memory, and other disorders that indicated frontal lobe dysfunction (1–3). These comorbidities severely impact quality of life, often more than the seizures themselves (4, 5). Understanding the nature of these deficits and finding effective treatment strategies is paramount.

We have previously shown that early life seizures (ELS) are associated with cognitive deficits. These deficits are often explored in the realm of hippocampal cognition (6, 7). More recently, we have also shown deficits in prefrontal cognition (8), which mirror deficits seen in children with epilepsy. Using both hippocampal- and prefrontal-dependent cognitive tasks [a water maze and a fear learning and extinction task that is associated with anxiety phenotypes in humans and animal models (9), respectively], we sought to understand the long-term effects of early treatment with ACTH and the corticosteroid dexamethasone on these aspects of cognition.

ACTH is used clinically for the treatment of infantile spasms. It reduces seizure frequency and may also have a positive impact of cognitive outcomes (10–14). This raises the possibility that ACTH could also be beneficial in other epilepsies associated with cognitive impairments. We have recently shown that ACTH is effective in ameliorating attention deficits associated with early life epileptiform activity and no seizures (15), but it remains uncertain whether ACTH could modify cognitive outcomes in the context of overt seizure activity that are not infantile spasms. Using an ELS model instead of an infantile spasms model allows us to explore the generalizability of ACTH treatment for other forms of pediatric epilepsy. We hypothesized that animals with a history of ELS would have significant deficits in fear conditioning and spatial cognition, and that ACTH administration during the time of the seizures would prevent these deficits. Dexamethasone is also used to treat some severe epilepsies in children (16). Dexamethasone and ACTH are often considered together as hormonal therapies given that a possible mechanism of action of ACTH is *via* stimulation of melanocortin 2 receptors in the adrenal with consequent release of corticosterone. However, if ACTH and dexamethasone are not similar in their effect on cognition associated with epilepsy, then it is possible that ACTH has mechanisms of action beyond stimulation of the adrenal.

MC2R is found solely in adrenal cortex, but all of the members of the melanocortin family of receptors bind ACTH with varying degrees of specificity. Two of these receptors, MC3R and MC4R, are expressed in the CNS, and therefore ACTH likely has direct CNS action in addition to its action on the adrenal. Recently, MC4R agonists have been used in animal models of Alzheimer’s disease and stroke as neuroprotective compounds that can also improve cognitive and behavioral outcomes (17–20). Although MC3R and MC4R agonism has been a suggested potential mechanism of action for ACTH in the treatment of seizures in infantile spasms (21), the role of the melanocortin system in cognitive impairment after ELS has yet to be explored. As such, the final aim of this study was to quantify expression of MCRs in the PFC and the hippocampus after ELS in order to begin to understand whether or not there is dysregulation of this receptor signaling pathway in our model.

The results show that ELS impairs PFC- and hippocampal-dependent cognition. Early administration of ACTH, but not dexamethasone, can prevent PFC-dependent cognitive impairment, suggesting dysregulation of central MCRs after ELS. Further supporting this hypothesis, MCRs are significantly downregulated in the PFC acutely after ELS. Importantly, this prevention occurs in the context of unchanged seizure outcomes.

## Materials and Methods

### Animals

All experiments were performed in accordance with the guidelines set down by the National Institutes of Health and the Geisel School of Medicine at Dartmouth for the humane treatment of animals. The animal protocol was approved by the Institutional Animal Care and Use Committee of Dartmouth College and the Institutional Animal Care and Use Committee of the University of Vermont.

### Early Life Seizure Model

Sprague-Dawley rats were subjected to a total of 62–65 flurothyl-induced seizures from postnatal day (p) 7 to p16. ELS rats and littermate controls were separated into three groups; vehicle treated, ACTH treated, and dexamethasone treated (*N* = 12 ACTH + gelatin vehicle, *N* = 14 dexamethasone + saline vehicle). Each rat received six to seven seizures per day and was allowed to recover for 1 h between seizures. Rat pups were placed into a sealed plastic container where 0.1 mL of flurothyl, an inhaled convulsive agent, was slowly dripped onto a small piece of filter paper within the chamber. Pups were observed carefully, and the chamber was unsealed and allowed to ventilate once animals showed signs of tonic forelimb and hindlimb extension. Littermate control pups were removed from the dam and handled at this time to control for the effect of handling- and maternal separation-related stress.

For experiments in which the effect of ACTH and dexamethasone on seizure latency and duration was examined, latency to seizure was observed in paired animals simultaneously to control for any discrepancy in injection volume and/or speed. Twenty-three rat pups (*N* = 9 ACTH, *N* = 10 vehicle, and *N* = 4 dexamethasone) were exposed to flurothyl in tandem (paired randomly together in the chamber). Latency to initial tonic forelimb extension and duration of seizure was timed in each animal. In this cohort of animals, only one seizure was administered per day for 5 days from p6 to p10 in order to assess changes in latency and duration over time.

### Drug Administration

For the ACTH fear learning/extinction task, ELS and littermate control animals were injected subcutaneously with 150 IU/m^2^ ACTH or the same dose of gelatin vehicle control, as previously described (10). Injections were made once daily, 1 h before ELS.

For the dexamethasone fear learning/extinction task, ELS and littermate control animals were injected subcutaneously with 0.5 mg/kg dexamethasone or the same volume of saline vehicle control. Injections were made once daily, 1 h before ELS.

The ELS paradigm was the same for both groups of rats. A subset of the animals was weighed at the time of behavior; adult weights do not differ as a function of treatment (*p* = 0.12 for ACTH treatment and *p* = 0.20 for dexamethasone treatment). Mortality in our study was low and unrelated to seizures or treatment in the majority of animals; only two vehicle-treated ELS animals, one ACTH-treated, and one dexamethasone-treated animal died during the seizure period.

### Water Maze

Animals were allowed to acclimate to the water maze room for 1 h before any testing began. Water maze was conducted in a large (2 m in diameter by 50 cm in height) tub filled with opaque water maintained at approximately 26°C. The room housing the water maze contained two distal cues placed on a black cloth covering the walls, approximately 50 cm away from the edge of the water maze. Indirect lighting was provided near the base of the water maze. On day 1, animals were allowed to habituate to the water and swim freely for 1 min without any platform present. A submerged platform was then introduced into the tub and testing began. Rats were placed tail-first into the water from four different starting locations selected randomly. Latency to find the platform was timed for four trials per day over the course of 4 days.

### Fear Learning Acquisition and Analysis

An operant box (Med Associates Inc., St. Albans, VT, USA) enclosed in a dimly lit, sound-attenuating chamber with a metal-bar floor was used for fear conditioning experiments. Rats were trained in a standard fear conditioning task. On day 1, all animals were habituated to the room and then the operant box where fear conditioning was to take place. On days 2 and 3, fear learning commenced. Each trial consisted of two 10 s presentations of a tone followed immediately by a 1-s, 0.75 mA footshock (inter-trial interval 64 s). There were three trials per session, one session per day. Behavior was recorded with a video camera and analyzed offline. For analysis, the instantaneous state of the rat was scored at 2, 10, 18, 26, 34, 42, 50, and 58 s after the second tone; 1 for moving and 0 for freezing (immobility with the exception of breathing). Freezing or moving scores were also collected during a 3-min pre-tone baseline in the operant chamber where the testing took place prior to administration of any tone or footshock.

### Fear Extinction

Fear extinction trials began 24 h after the second session of fear learning. For extinction, rats were placed in the same operant box, and the conditioned stimulus tone was played 30 times for 10 s. Behavior was recorded with a video camera and analyzed offline. Moving behavior at 2, 6, 8, and 10 s after the beginning of the tone was scored and averaged to give one value per tone; 1 for moving and 0 for freezing at each of those time points. Freezing was defined as complete immobility with rigid body posture.

### Immunohistochemistry and Cell Counting

Early life seizures animals (*N* = 8 control, *N* = 9 ELS) were euthanized at p19, 36 h after the ELS paradigm had ended. Animals were deeply anesthetized with isoflurane (4–5% in oxygen) until breathing slowed, and rats were unresponsive to a tail and paw pinch. Animals were then perfused transcardially with approximately 20 mL of PBS followed by 30 mL of 4% paraformaldehyde. Brains were then post-fixed in 4% paraformaldehyde overnight (18 h) and transferred to 30% sucrose until the point of sinking (2–3 days). Brains were placed in optimal cutting temperature (OCT solution) and flash-frozen at −80°C. Coronal sections through the infralimbic cortex and dorsal hippocampus were cut on a −25°C cryostat at 30 μm and placed into a 24-well plate containing 1 mL wells of PBS.

Endogenous peroxidase activity was then blocked with 1 mL 3% hydrogen peroxide for 5 min, followed by three 5-min washes in PBS. Non-specific antigen binding was blocked by 600 μL of 1% BSA, 3% normal goat serum, and 0.25% Triton X-100 in PBS for 30 min on a rocker. The sections were then incubated with 400 μL of either 1:400 rabbit Anti-Melanocortin Receptor 3 (extracellular) or 1:700 rabbit Anti-Melanocortin Receptor 4 (extracellular) antibodies (Alomone Labs, Jerusalem, Israel) at 4°C overnight (18 h) on a rocker. This was followed by three 5-min washes in PBS, followed by incubation with 400 μL 1:1000 Biotinylated Goat Anti-Rabbit IgG Antibody (Vector Labs, Burlingame, CA, USA) for 75 min at 25°C on a rocker. Five hundred microliters of ABC avidin–biotin solution (Vector Labs) were then added to wells, and the plate was placed on a rocker for 30 min. Sections were washed three times with PBS, before 400 μL of DAB–peroxidase substrate (Vector Labs) was added and left for 2–4 min. Sections were washed three times in PBS, then mounted onto charged slides, and dehydrated sequentially in baths of 70, 95, and 100% ethanol for 5 min each. Sections were cleared in methyl salicylate for 5 min, before coverslips were mounted using toluene-based mounting medium, and sealed with clear nail polish.

For cell counting, we used a microscope with Koehler illumination at 20× magnification to count cells in layers 2–3, 5, and 6 of the infralimbic cortex, and in the CA1 pyramidal cell layer of the hippocampus. About 20× magnification was used because it allowed for identification of individual cells and the decreased zoom allowed for use of a larger defined counting frame. Eight coronal sections from each brain region were taken from each rat and stained according to the above method: four with MC3R antibody and four with MC4R antibody. Collection for PFC began based on visually identified corpus callosum landmarks at coordinates +3.5 mm to +2.9 mm anterior to bregma. Collection for hippocampal sections began based on ventricular and cornu amonnis landmarks at coordinates −2.8 to −4.0 mm posterior to bregma. Every third PFC section and every sixth hippocampal section were collected and stained. We used the optical fractionator function of Stereo Investigator (MBF Bioscience, Williston, VT, USA), with unbiased counting frames of 50 μm × 50 μm in the PFC and 25 μm × 25 μm in the hippocampus and grid sizes of 200 μm × 200 μm and 100 μm × 100 μm, respectively. The optical fraction was set at 10 μm depth, centered in the middle of the tissue section encompassed by the counting frame. The optical fraction was realigned for every new counting frame. Only cell tops falling within the optical fraction were counted. Cells touching the red line of the counting frame were discounted, whereas those touching the green line were included in the count. A total of 1210 control and 1238 ELS cells were counted in CA1 over an average volume of 1,990,302.6 ± 164,005.6 μm^3^ in controls and 1,773,171.4 ± 101,309.4 μm^3^ in ELS animals; a total of 16,833 control and 8193 ELS cells were counted in mPFC over an average volume of 5,062,576.6 ± 188,003.2 μm^3^ in controls and 3,605,728.1 ± 133,541.2 μm^3^ in ELS animals. Cells were counted blindly to reduce subjective bias. To eliminate bias due to area and thickness of tissue, cell counts were divided by volume of the section to obtain the more representative measure of cell density.

### Statistics

All fear learning and extinction data and cell staining counts were modeled using generalized estimating equations (GEE) in SPSS v22 (IBM corporation, Chicago, IL, USA) in order to ensure that the most appropriate data distribution was assumed and in order to deal with repeated measures. For the behavioral data, the dependent variable was moving score. For the immunohistochemistry (IHC) data, the dependent variable was number of cells stained per volume of tissue. Group was modeled as a factor, with tone number as a covariate in the behavioral data. Fear extinction moving scores were best modeled as a linear scale response, whereas fear learning scores were modeled assuming a binomial distribution. In order to understand how the learning differed between the groups, all data were examined as group by tone effects and therefore describe mean differences between groups in the rate of learning as a function of tone. For extinction data where differences in the 30th (final) tone were reported, a one-way ANOVA using a Tukey *post hoc* test was used. Seizure duration and latency were also modeled using a GEE assuming a gamma distribution. Treatment (ACTH or vehicle) was used as a factor with day as a covariate.

Morris water maze data were analyzed using a multivariable Cox regression time-to-event approach in STATA (SEv13, StataCorp, TX, USA), as previously described (22, 23). Traditional analyses used to analyze water maze data have been questioned and time-to-event analyses are considered to be more appropriate. Specifically, Jahn-Eimermacher et al. argue against the use of ANOVAs, etc., in lieu of survival analysis for water maze and other behavioral data (24). Frailty Cox models were applied to deal with repeated measures over day. In addition to being the most appropriate way to analyze water maze data, this approach also allowed us to investigate the interaction between ELS and ACTH treatment while accounting for within-animal correlations. The time-to-event was the latency (in seconds) to find the platform.

All values are reported as estimated marginal means ± SEM.

## Results

### ELS Cause Significant Deficits in Fear Extinction but Not Fear Learning

**Figure 1:**
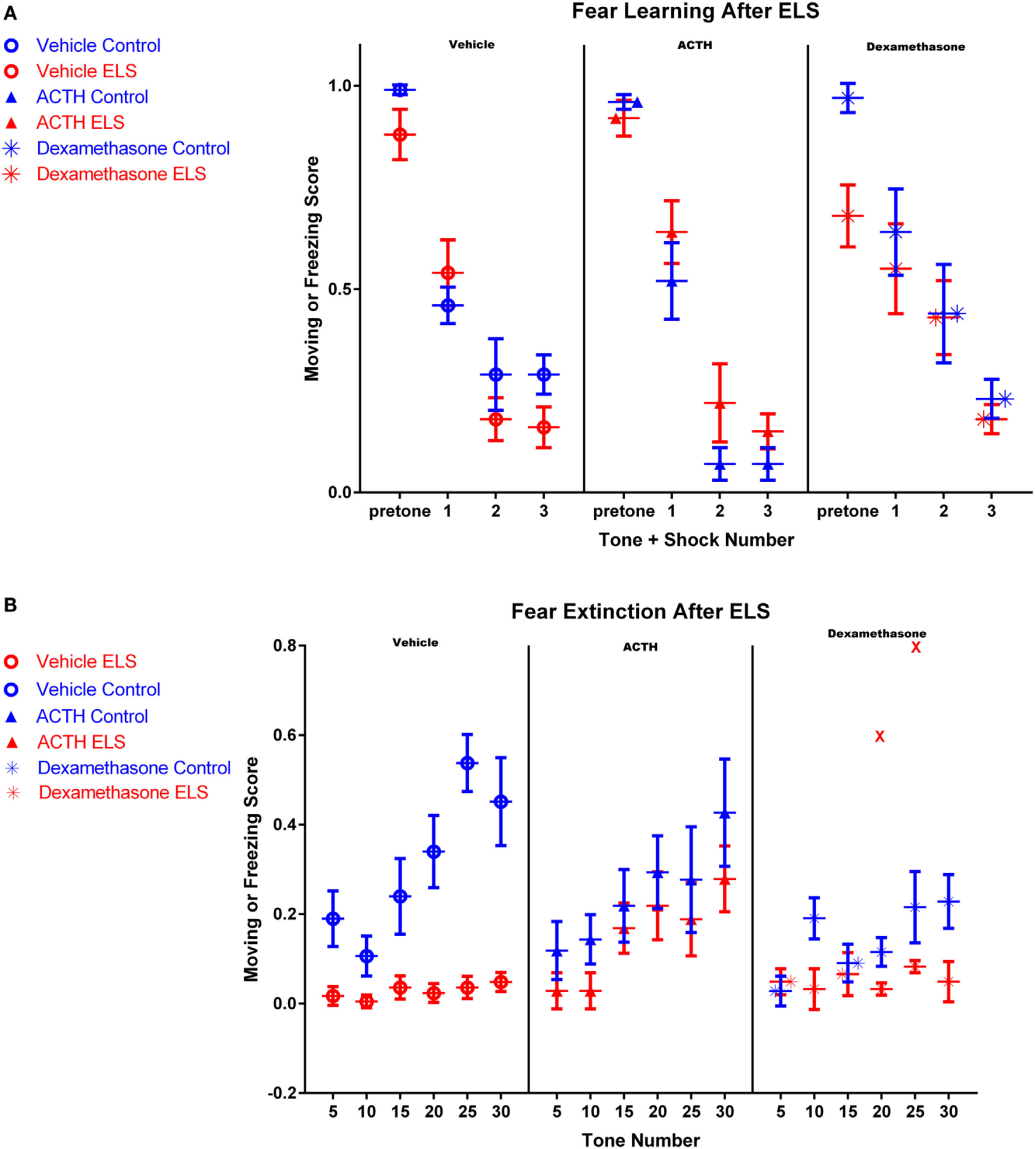
**Fear conditioning after ELS and treatment with dexamethasone or ACTH**. Each panel represents a treatment condition, with the left panel depicting moving scores from vehicle-treated animals, the middle panel showing ACTH-treated animals, and the right panel dexamethasone-treated animals. All animals in all groups acquired the fear learning paradigm after three tone-shock pairings **(A)**. However in **(B)**, vehicle-treated ELS animals (red open circles, left panel) had significant deficits extinguishing the fear task compared to vehicle-treated controls (blue open circles, left panel), even after 30 tones without shock pairing. Treatment with ACTH (filled triangles, middle panel) did not alter fear extinction in control animals (blue) but significantly prevented fear extinction in animals with a history of ELS (red). Treatment with dexamethasone (stars) did not significantly improve fear extinction in animals with a history of ELS (red), even with the inclusion of one outlier animal representing two data points at tones 20 and 25 (shown as red Xs in the right panel).

We first tested the effect of ELS on fear learning acquisition and subsequently fear extinction, two prefrontal-dependent tasks. No significant alterations in fear learning were noted in vehicle-treated animals experiencing ELS (*N* = 8) compared to controls (*N* = 8) (ELS effect; *p* = 0.427). ACTH administration in control (*N* = 6) or ELS animals (*N* = 5) did not significantly affect fear learning (Figure 1A) and nor did dexamethasone administration in control (*N* = 4) or ELS animals (*N* = 3) (Figure 1A) (main effect of treatment; *p* = 0.575). Overall, all groups of animals learned the task in one session (main effect of tone; *p* < 0.001).

Twenty-four hours after the end of fear learning trials, fear extinction trials began. ELS rats had a significant deficit in fear extinction and exhibited freezing behavior significantly more frequently throughout the whole session than controls (Figure 1B; *p* < 0.0001). Although the vehicle-treated control animals exhibited clear extinction over the session, the vehicle-treated ELS group had a moving score of 0.05 ± 0.02 by the 30th tone, where a value of 1 would indicate moving during all measured time points after the tone and a value of 0 would indicate freezing during all measured time points after the tone. This demonstrates that ELS animals were unable to extinguish even at the end of the extinction session (Figure 1B). In addition, moving scores of vehicle-treated ELS animals were significantly different to vehicle-treated controls at the 30th tone (*p* < 0.001).

### ACTH Improves Fear Extinction in ELS Animals

The rate of fear extinction is not altered with ACTH administration in control animals (*p* = 0.45; group by tone, vehicle-treated controls vs. ACTH-treated controls). However, importantly, the rate of extinction in ELS animals treated with ACTH is also indistinguishable from vehicle-treated controls (*p* = 0.15; group by tone, vehicle-treated controls vs. ACTH-treated ELS, see Figure 1B). This suggests that ACTH restores fear extinction in animals with a history of ELS. This is also reflected by comparison of moving scores at the end of the fear extinction session. At the 30th tone, moving scores of ACTH-treated ELS animals were significantly higher than ELS animals (*p* = 0.006) and were not significantly different from vehicle-treated controls (*p* = 0.1).

### ACTH Does Not Ameliorate Water Maze Deficits

**Figure 2:**
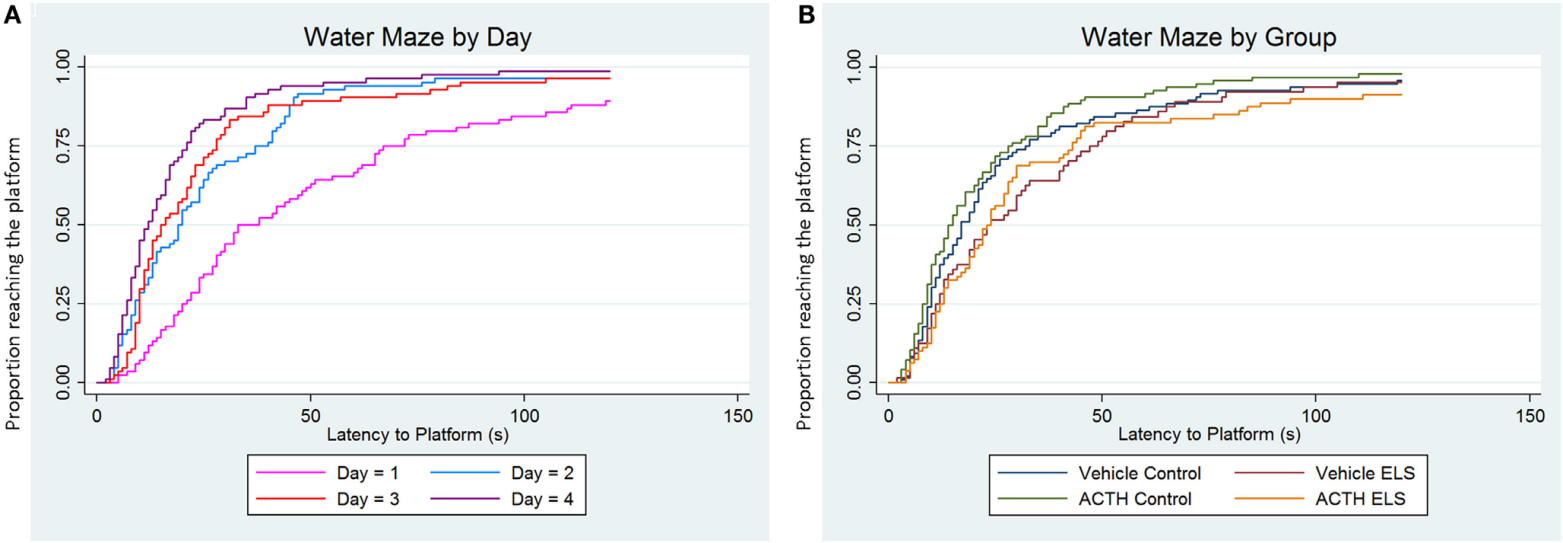
**ELS-associated deficits in water maze are not improved with ACTH**. Time-to-event (survival) analysis of the latency to find the platform in a Morris water maze task show that animals have a shorter latency to find the platform on day 4 (purple line) compared to day 1 (pink line). This indicates that, when adjusted for group, animals on average learn over the 4-day time course of the experiment **(A)**. We then show in **(B)** that animals with a history of ELS have significant deficits in water maze (green line) over controls (blue line), seen as an increased proportion of animals having longer latencies to find the platform when day and trial are accounted for statistically. Treatment with ACTH during the time of the seizures does not improve this deficit (yellow line). Likewise, treatment with ACTH does not alter latency to find the platform in control animals (red line) as well.

We have previously reported that the ELS paradigm significantly impairs hippocampal-dependent learning and memory. In order to determine whether or not treatment with ACTH was effective in ameliorating these deficits, we used a second cohort of animals that had experienced ELS with (*N* = 5) or without (*N* = 4) ACTH treatment from p6 to p16. Overall, all animals learned to find the platform, as seen by a significant effect of day (*p* < 0.001, Figure 2A). As previously reported, animals experiencing ELS have significant impairments during the Morris water maze (*p* = 0.01) in adulthood compared to controls (*N* = 6 treated with vehicle; *N* = 6 treated with ACTH). We found no change in water maze performance when animals were administered ACTH (*p* = 0.71) suggesting that treatment with ACTH does not improve hippocampal-dependent spatial cognition (Figure 2B).

### Dexamethasone Does Not Improve Fear Extinction in ELS Animals

Early treatment with dexamethasone may actually worsen the rate of extinction for control animals (*p* = 0.003, group by tone effect comparing vehicle-treated to dexamethasone-treated controls), although the groups are not significantly different by the end of the extinction session (*p* = 0.07 at tone 30).

However, unlike ACTH, dexamethasone treatment in ELS animals does not restore fear extinction to the levels of vehicle-treated controls as the rate of extinction was significantly different between the groups (*p* = 0.04; group by tone comparing vehicle control to dexamethasone-treated ELS). It is also worth noting that one animal in this group had outlier measurements at tone 20 and tone 25 (shown as red Xs in panel 3 of Figure 1B); with these outliers removed, this effect is even more significant (*p* < 0.001). Indeed, dexamethasone treatment did not significantly improve the rate of fear extinction compared to vehicle-treated ELS (*p* = 0.39, group by tone comparing vehicle-treated ELS to dexamethasone-treated ELS). This is also true when comparing moving scores at the final tone (*p* = 0.966, group effect comparing vehicle-treated ELS to dexamethasone-treated ELS).

### ACTH and Dexamethasone Do Not Alter Seizure Latency or Duration

**Figure 3:**
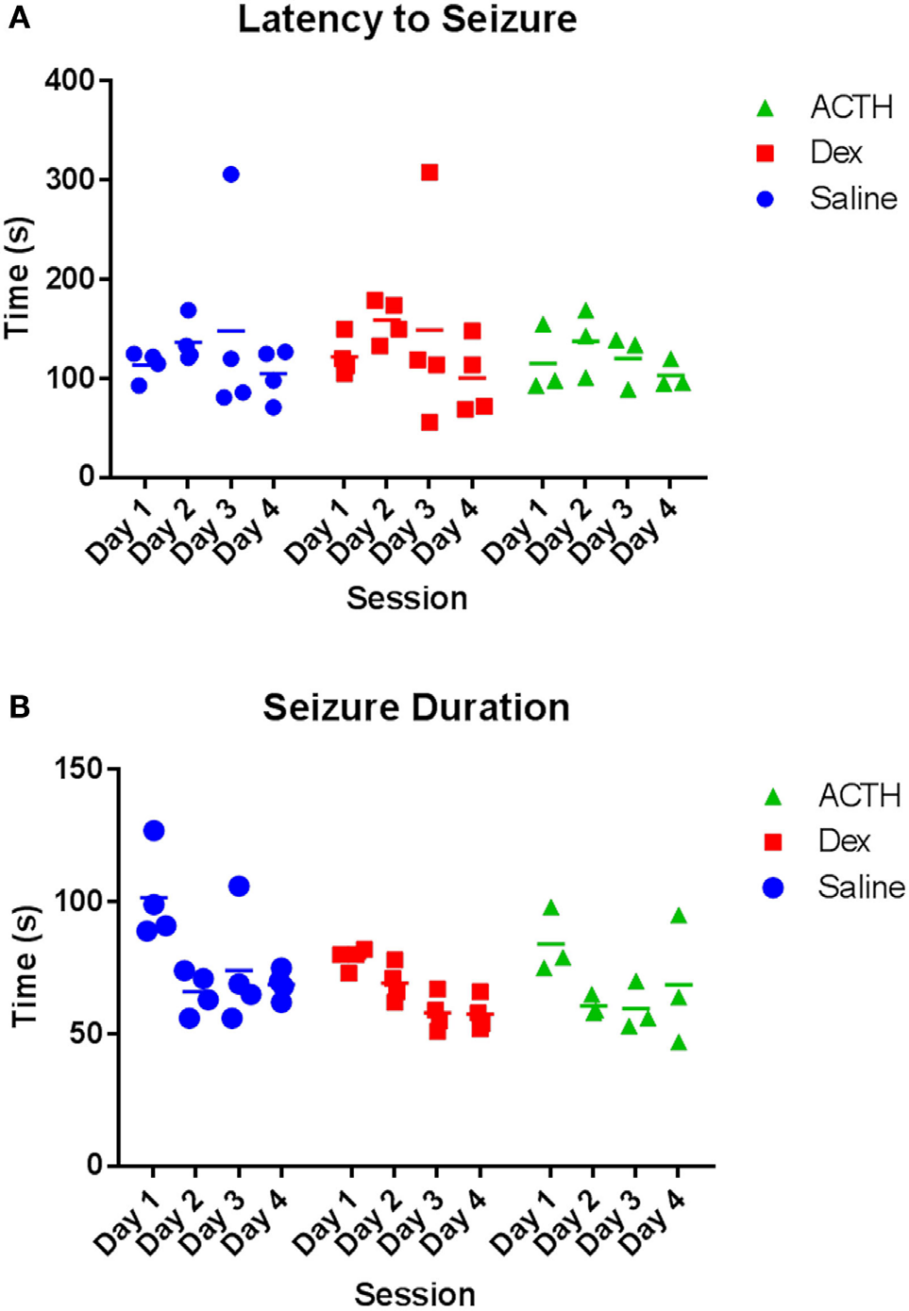
**Seizure parameters are not altered by dexamethasone and ACTH treatments**. Treatment with dexamethasone (squares) or ACTH (triangles) does not alter latency to flurothyl seizure or latency to flurothyl seizure over the course of 4 days compared to vehicle-treatment (circles) **(A)**. Likewise, treatment with dexamethasone or ACTH did not significantly alter seizure duration **(B)**.

Having found that ACTH partially rescued the ability of ELS rats to extinguish fear, we then wanted to determine the effects of ACTH and dexamethasone administration on seizure properties in the flurothyl model of ELS. Overall, we found a significant reduction in latency and an increase in duration in both groups over day (main effect of day: *p* = 0.004 duration of seizure; *p* < 0.001 latency to seizure). There was no difference in the latency (Figure 3A) to or duration (Figure 3B) of flurothyl-induced seizures with administration of ACTH or dexamethasone; mean latency was 116.47 ± 11.5 s in the vehicle-treated animals compared to 106.29 ± 5.6 s in ACTH-treated animals and 119.16 ± 18.5 s in dexamethasone-treated animals (*p* = 0.63 group effect; *p* = 0.98 group × day effect), mean duration was 80.24 ± 12.6 s in vehicle-treated animals compared to 66.71 ± 4.9 s in ACTH-treated animals and 68.52 ± 7.8 s in dexamethasone-treated animals (*p* = 0.44 group effect; *p* = 0.27 group × day effect) (Figure 3).

### ELS Significantly Reduce MC3R and MC4R Expression in the PFC but Not in CA1

**Figure 4:**
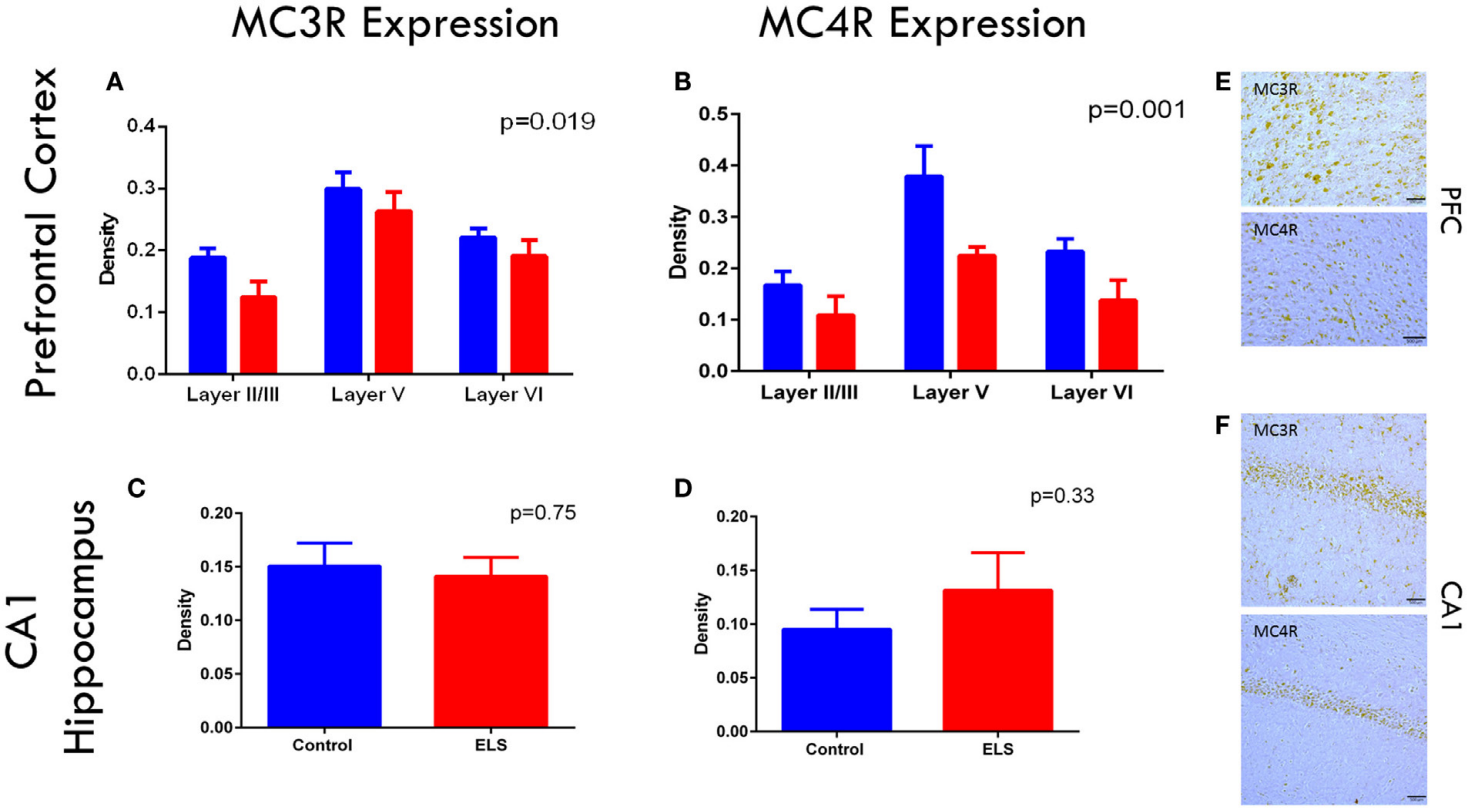
**Melanocortin receptor expression is downregulated in the PFC after ELS**. In all layers of the mPFC, MC3 **(A)** and MC4 **(B)** receptors are significantly downregulated (*p* = 0.019 for MC3Rs; *p* = 0.001 for MC4Rs) in ELS animals (red bars) compared to control (blue bars). No significant group by layer effect was noted. No significant changes were noted in CA1 of the hippocampus in either MC3 **(C)** or MC4R **(D)** expression after ELS. Representative images of MC3R and MC4R staining in PFC are shown in **(E)**, and MC3R and MC4R staining in CA1 are shown in **(F)** at 20× magnification. *N* = 8 control, *N* = 9 ELS.

As ACTH only improved the rate of fear extinction in animals with a history of ELS and dexamethasone did not, we wanted to determine whether the melanocortin receptor system of the CNS was altered after ELS. If central MCR system dysfunction was noted after ELS, this could point to a role of MCRs in the mechanism of action of ACTH in improving cognitive outcome. To address this question and confirm the presence of these receptors in these brain regions at this age, we stained and quantified cells expressing MC3R and MC4R in the infralimbic cortex of the mPFC and CA1 of the dorsal hippocampus in control and ELS animals at p19 (Figures 4A–F). In the infralimbic cortex, we found that the density (cell count/10,000 μm^3^) of cells expressing these MCRs was significantly lower in ELS animals than in controls across all layers (Figures 4A,B; *p* = 0.019 for MC3R; *p* = 0.001 for MC4R). However, the density of cells expressing MC3R or MC4R in the pyramidal cell layer of CA1 was not significantly different between groups (Figures 4C,D; *p* = 0.75 for MC3R; *p* = 0.33 for MC4R).

## Discussion

Children with many different forms of epilepsy are significantly more likely to experience cognitive deficits consistent with frontal lobe dysfunction, including anxiety (25–28). Fear extinction is a reliable measure of prefrontal cognition in animal models (29, 30). Here, we show that rats with a history of ELS have significant deficits in fear extinction and spatial learning as adults, confirming that ELS animals have both hippocampal and prefrontal dysfunction. Additionally, we show that ACTH can prevent some of these cognitive deficits. This is the first time that the long-term cognitive impact of treatment with ACTH has been shown and suggests that add-on treatment with a melanocortin agonist during childhood seizures may lead to improvements in specific cognitive domains during adulthood. It is worth noting that the decision to use an ELS model *instead* of an infantile spasms model allows us to understand the generalizability of ACTH treatment for other forms of pediatric epilepsy as well.

Treatment with either ACTH or dexamethasone 1 h prior to seizures did not significantly reduce seizure duration or latency in our model; but interestingly ACTH, and not dexamethasone, was able to prevent deficits in fear extinction in the long term. This suggests that ACTH has an additional mechanism to its cortisol-mediated effect, which could prevent deficits in prefrontal cognition. This also serves to challenge the notion that seizures themselves are solely responsible for cognitive deficits – a notion that pervades much of the literature as an unproven assumption. If ACTH does not alter seizure parameters and seizures were the sole cause of cognitive deficits, we would not expect ACTH to positively impact cognition, as shown both here and in a different seizure-related model (15), it does. To the best of our knowledge, only one other group has data to suggest that cognitive and seizure outcomes may be dissociable. In a model of prolonged febrile seizure, decreased T2 signal in the whole brain is neuroprotective in terms of cognitive outcome (31), but decreased T2 signal in the amygdala is a biomarker for the development of future temporal lobe epilepsy (32). An improvement in cognitive outcome in the context of a negative seizure improvement outcome has never been evaluated in humans; our data presented here and the data of Baram’s group suggest that this may warrant further study.

An unexpected finding of our research was that ACTH was able to ameliorate cognitive deficits in PFC-dependent fear extinction, but it was unable to do so in hippocampal-dependent spatial learning. This difference in drug efficacy between the two tasks suggests that following ELS there may be something fundamentally different in the way these two brain regions respond that enables ACTH to protect one, but not the other. Quantification of MC3R- and MC4R-expressing cells reveals a correlation that may pose an explanation. Our results show that MC3R and MC4R are expressed in both the infralimbic cortex and in the CA1 of hippocampus in control rats. However, following ELS, the density of cells expressing MC3R and/or MC4R was significantly decreased in the PFC compared to controls, but *not* significantly different in the CA1 between ELS rats and controls. As ACTH binds these receptors and was able to protect PFC cognition but not hippocampal cognition, this suggests a role for central melanocortin signaling in this cognitive protection. We initially chose a time point of 36 h after the induction of the last seizure in order to assess the acute effect of the ELS insult on receptor expression. Our results show that MCRs are present in mPFC and CA1 at p19 and are dysregulated by the ELS insult, thereby strengthening the hypothesis that ACTH acts *via* a CNS MCR-mediated mechanism to protect against injuries to prefrontal cognition. Important future directions include characterization of the effect of ACTH on the expression of MC3R and MC4R in rats with a history of ELS, and whether these differences in expression between control and ELS rats at p19 persist into later life.

ACTH, acting through MC2Rs in the adrenal cortex, leads to systemic release of glucocorticoid steroid hormones; and although glucocorticoid steroid hormones, such as dexamethasone, are able to modulate the formation, consolidation, and retrieval of fear memory acutely in adult controls (33), their long-term effect on pediatric patients with a history of seizures is unknown. The comparison of ACTH and dexamethasone in our study suggests that typical glucocorticoids would not have the same positive long-term impact on patients as ACTH, despite acute administration of glucocorticoids enhancing extinction in adults with phobias (34), and exogenous corticosterone administration facilitating fear extinction in mice (35). Indeed, an unexpected finding of the current study was the suggestion of a detrimental effect of dexamethasone on control rats. While the mechanism for this is unknown and not within the scope of the current study, it does indicate that the positive impact of ACTH on cognition is not entirely due to corticosteroid release. This also suggests that ACTH and not prednisone, another glucocorticoid drug given to patients that has some mineralocorticoid activity but no activity at melanocortin receptors, may be beneficial in improving cognitive outcome. This result is not entirely unexpected, as there is some evidence that treatment with ACTH can improve cognitive outcomes in human patients with epilepsy. In a study done by Kivity and colleagues, children with cryptogenic infantile spasms normally associated with poor cognitive outcome were treated with high-dose ACTH. The authors note that early treatment was associated with a favorable long-term cognitive outcome in all patients (10). In the UKISS study evaluating treatments for infantile spasms, children with no identified etiology and who received either ACTH or a corticosteroid, had significantly higher Vineland adaptive behavior scores at follow-up than children receiving vigabatrin (11). Interestingly, in parallel to our current study, no clear correlation between seizure freedom and outcome was established in the UKISS study. We show here for the first time a potential mechanism for this improvement. Together, this research suggests that early diagnosis and treatment of pediatric epilepsies with ACTH may be of utmost importance to avoid some of the associated deleterious cognitive dysfunction.

Our results provide the first investigations of a potential mechanism underlying ACTH-related cognitive improvement after ELS, potentially implicating central melanocortin receptors, MC3 and/or MC4R. Understanding the mechanism behind improved cognitive outcome has the potential to elucidate novel therapeutic targets for specifically directed add-on therapies for cognition in epilepsy. Additionally, the effectiveness of ACTH in improving cognitive outcome in our ELS model, instead of an infantile spasms model, suggests potential for generalizability of this putative novel therapeutic target beyond very severe epilepsies more broadly to pediatric epilepsy in general.

## Author Contributions

AH, GH, and RS designed the study; AH, AM, and DL acquired and analyzed the data; AH, AM, and RS drafted the manuscript and figures.

## Conflict of Interest Statement

The authors declare that the research was conducted in the absence of any commercial or financial relationships that could be construed as a potential conflict of interest.
